# Chemokines and chemokine receptors in Behçet’s disease

**DOI:** 10.3389/fimmu.2023.1109147

**Published:** 2023-01-18

**Authors:** Zhan Li, Linlin Cheng, Haoting Zhan, Yongzhe Li

**Affiliations:** ^1^Department of Clinical Laboratory, Peking Union Medical College Hospital, Chinese Academy of Medical Science and Peking Union Medical College, Beijing, China; ^2^State Key Laboratory of Complex, Severe and Rare Diseases, Peking Union Medical College Hospital, Chinese Academy of Medical Science and Peking Union Medical College, Beijing, China

**Keywords:** Behçet’s disease, chemokine, chemokine receptor, cell infiltration, therapeutic target

## Abstract

Behçet’s disease (BD), a chronic vascular inflammatory disease, is characterized by the symptoms of ocular lesions, recurrent genital and oral ulcers, skin symptoms and arthritis in addition to neurological, intestinal and vascular involvement. The pathogenesis of BD is poorly understood, and there are no effective laboratory markers for the diagnosis of BD. In addition, BD is presently incurable. Chemokines, a family of small secreted chemotactic cytokines, interact with chemokine receptors and mediate the migration, localization and cellular interactions of inflammatory cells. Several studies have suggested that chemokines and their receptors play an important role in the occurrence and development of BD and that these chemokines along with their receptors can be utilized as biomarkers and therapeutic targets. In the present review, chemokines and chemokine receptors involved in BD and their potential application in diagnosis and therapy have been discussed.

## 1 Introduction

Behçet’s disease (BD) is a recurrent vascular disorder that affects multiple systems, with the highest prevalence found along the ancient silk road from the Mediterranean *via* the Middle East to East Asia and an estimated prevalence of 14 per 100,000 people in China (1, 2). The pathogenesis of BD remains unclear, with genetically susceptible background and environmental and microbial risk factors triggering inflammatory and immunologic responses (3, 4). These responses collectively contribute to the initiation of the disease. Inflammation in BD is characterized by the infiltration of several inflammatory cells, including natural killer (NK) cells, neutrophils and T lymphocytes (5). BD is usually diagnosed in accordance with clinical manifestations, organ involvement, pathergy test and histopathology (6). Common strategies recommended for the management of BD depend on the involvement of the specific organ; BD is managed by molecules such as colchicine, glucocorticoids, apremilast, interferon (IFN)-α, tumor necrosis factor (TNF)-α inhibitors, and certain immunosuppressive agents, including azathioprine, thalidomide, cyclosporine-A and cyclophosphamide (7). However, BD is incurable, and the management of patients with refractory BD symptoms is challenging (8).

Chemokines are a superfamily of small, secreted proteins that facilitate the migration of immune cells into inflammatory sites by interacting with their receptors. This process plays a central role in the occurrence and development of several autoimmune diseases, including rheumatoid arthritis, systemic sclerosis and systemic lupus erythematosus (9). For instance, C–C motif chemokine ligand (CCL)18 has been suggested to be an appropriate biomarker that reflects immunoglobulin G4-related disease activity and therapeutic efficacy (10), whereas CXC chemokine receptor (CXCR)7 has been shown to aggravate autoimmune encephalomyelitis by increasing the migration of leukocytes into the central nervous system parenchyma in a multiple sclerosis model by scavenging CXCL12 (11). In the past two decades, several chemokines and their receptors associated with BD have been reported (12, 13). Chemokines and their receptors participate in the pathogenesis of BD by mediating four major processes: the regulation of gene susceptibility, chemotaxis, activation of immunocytes and cascade amplification of the inflammatory response. The expression levels of chemokines and chemokine receptors are often significant in patients with BD, suggesting their potential application in laboratory diagnosis and therapeutic effect evaluation. Herein, an overview of chemokines and chemokine receptors that are associated with the pathogenesis of BD and their value and challenges as biomarkers and therapeutic targets is provided.

## 2 Chemokines and chemokine receptors as mediators

Accumulating evidence has shown that certain chemokine and chemokine receptor genes can increase or decrease the risk of developing BD through gene susceptibility and that the genetic background of BD varies among different gender, regions and ethnicities. A Turkish study reported that CXCL8 gene polymorphisms possibly affect susceptibility to BD in people from the Denizli Province (14). The study revealed that the CXCL8 −251 TT genotype increases susceptibility to BD in males and the −251 AA genotype increases susceptibility in females. In addition, the −251 TT genotype and T allele are associated with ocular involvement and the −251 AA genotype is associated with erythema nodosum; however, −251 AT was shown to be protective against BD. Another Turkish study revealed that the CXCL5 rs352046 (−156, G>C) polymorphism and CXCR2 rs2230054 TT genotype increase the risk of developing BD (15). In the Chinese Han population, the AA genotype of monocyte chemoattractant protein-1-2518 (rs1024611/CCL2) was associated with protection from developing ocular BD and the AG genotype showed increased susceptibility toward the same (16). In addition, studies have shown that the C–C chemokine receptor (CCR)5 Δ32 polymorphism increases the risk of BD in Iranian women and in both Italian men and women but not in the British, Turkish, Palestinian and Portuguese population (17–19). A meta-analysis also confirmed the risk factor of CCR5 Δ32 allele in the development of BD among people with the HLA-B51 allele (20).

**Figure 1:**
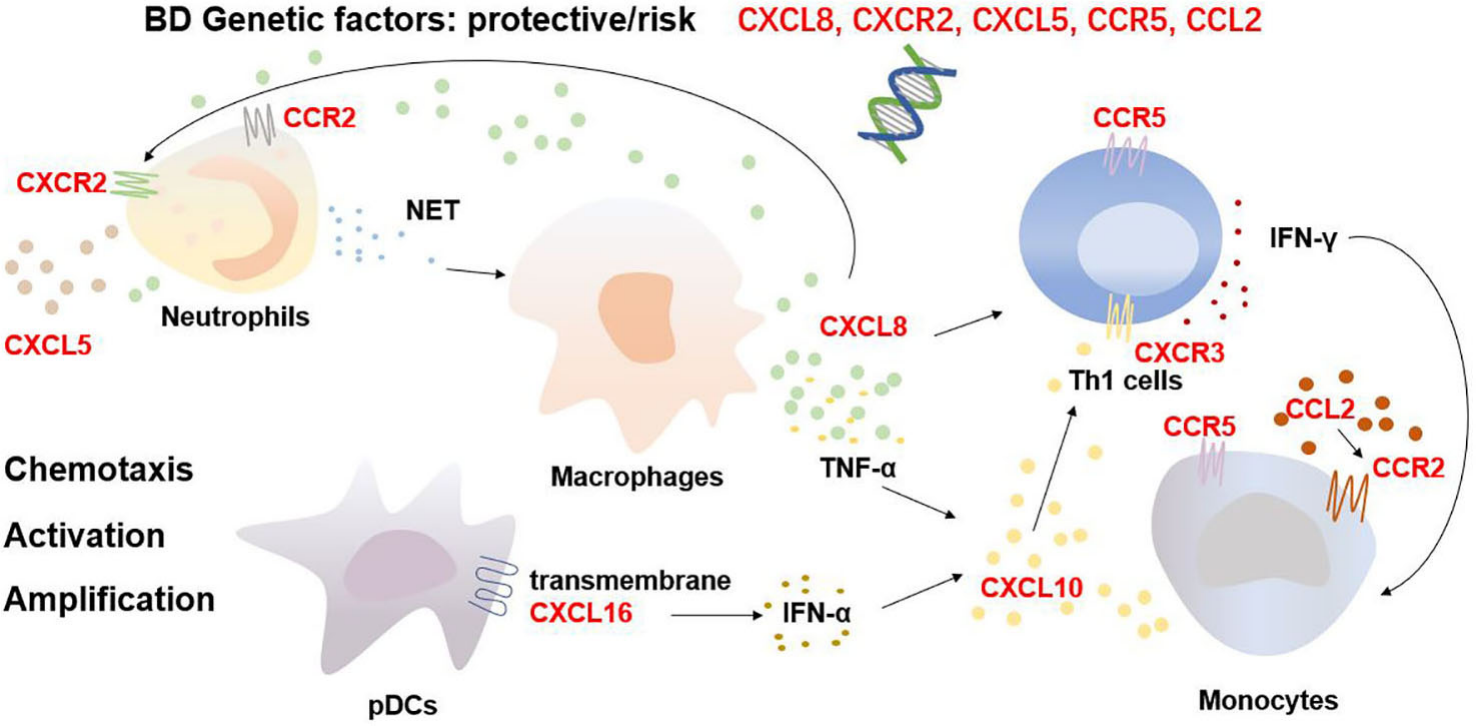
BD inflammatory microenvironment mediated by chemokines and chemokine receptors.

Besides genetic susceptibility, chemokines and their receptors mediate the migration and activation of immune cells as well as provoke the cascade amplification of inflammatory responses, thereby initiating and maintaining BD progression **(** **Figure 1****)**. As the critical cellular participants in the pathogenesis of BD, overactivated neutrophils release abundant reactive oxygen species *via* nicotinamide adenine dinucleotide phosphate hydrogen oxidase and neutrophil extracellular traps (NETs), inducing thrombo-inflammation (21, 22). NETs also contribute to the death of endothelial cells and are associated with the BD skin lesions of vasculitis and panniculitis (23). CXCL8, CXCL5, CXCR2, and CCR2 promote the recruitment and activation of neutrophils. Chemokines also hasten the development of BD *via* a positive feedback loop. In BD, neutrophils release more NETs, which stimulate macrophages to produce CXCL8 and TNF-α. The released CXCL8 attracts and activates more neutrophils and enhances the adhesion of neutrophils to endothelial cells, amplifying the cascade (24, 25). In addition, Th1 cells and monocytes are involved in the pathogenesis of BD. Th1 cells, as per their function, dominate BD inflammation by releasing Th1 cytokines such as IFN-γ and TNF-α (26), and the impairment of M2 monocyte/macrophage-mediated anti-inflammatory function and M1 predominance are crucial in the development of BD uveitis and neuroinflammation (27). CXCL10, CCR5 and CXCR3 are involved in the migration and infiltration of Th1 cells, whereas CCL2, CCR2 and CCR5 recruit monocytes into BD inflamed sites. CXCL8 reportedly accelerates the production of IFN-γ from Th1 cells as a cofactor/costimulatory molecule and promotes the differentiation of IFN-γ^+^ CD4^+^ T cells (28). Furthermore, CXCR3 potentially promotes the differentiation of naïve T cells to Th1 cells by interacting with CXCL10 (29), whereas transmembrane CXCL16 on the surface of plasmacytoid dendritic cell may promote high serum IFN-α expressions (30). IFN-γ, TNF-α and IFN-α facilitate the release of CXCL10 partially from monocytes to mediate the migration and infiltration of additional Th1 cells (31), potentially constituting another positive feedback loop. In general, the chemokine family influences the migration and localization of immune cells, thereby contributing to cellular infiltration as well as the inflammatory microenvironment in patients with BD.

## 3 Chemokines and chemokine receptors as biomarkers

Significant differences in the levels of some chemokines and chemokine receptors in patients with BD compared with healthy individuals and in patients at the different stages of BD underline their potential as biomarkers for diagnosis, disease activity reflection and therapeutic effect evaluation (**Table 1**). Increased CXCL8 levels have been detected in the serum, aqueous humor, cerebrospinal fluid and synovial fluid of patients with BD (32–35). CXCL8 has been suggested as a biomarker for BD activity as its expression is associated with the duration of symptoms, time elapsed since the primary diagnosis and recurrence of BD manifestations (36, 37). CXCL10 levels are also elevated in the aqueous humor and cerebrospinal fluid of patients with BD (31, 33), and serum CXCL10 levels have been correlated with mucocutaneous lesions and BD activity (38). CXCL1 is believed to be a BD biomarker with high sensitivity and accuracy, and may be useful in BD diagnosis and therapeutic effects. Indeed, it is found in abundant levels in the serum and aqueous humor of patients with BD, and its expression has been correlated with the disease activity of BD (39, 40). CCL4, another possible biomarker (the area under the curve = 0.734~0.823), can detect the BD inflammatory status; higher CCL4 mRNA expression has been observed in patients with BD along with central nervous system involvement and thrombosis, leading to the migration of additional CD8^+^ T cells, activated mast cells, M1 macrophages and NK cells to the inflammatory sites and aggravating the disease activity (41). CCL2 has also been shown to be elevated in the serum, plasma and whole blood of patients with BD (32, 42). Moreover, it has been observed that the levels of CCL3 are elevated in the serum of patients with BD, and negatively correlated with the levels of CCL5 (43).

**Table 1:** Chemokines and chemokine receptors with potential for BD laboratory biomarkers.

Potential Biomarker	Variation	Sample type	Manifestations	Clinical relevance
Chemokines				
CXCL8	Elevated	Serum		Duration, recurrence
CXCL10	Elevated	Aqueous humor, cerebrospinal fluid	Mucocutaneous lesions	Activity
CXCL1	Elevated	Serum, aqueous humor		Diagnosis, therapeutic effects, activity
CCL4	Elevated		Central nervous system involvement, thrombosis	Activity
CCL2	Elevated	Serum, plasma, whole blood		
CCL3	Elevated	Serum		
Chemokine receptor				
CXCR3	Elevated Inverse correlation with CXCL10	CD3^+^ T cells	Pulmonary involvement, central nervous system involvement	
CCR5	Elevated	Tissue, peripheral blood lymphocytes		
CXCR2	Elevated	Leukocytes, neutrophils	Ocular involvement	Activity

The expression of the chemokine receptors CXCR3, CCR5 and CXCR2 have been observed to vary in BD. CXCR3 predominantly recruits Th1 cells by interacting with CXCL10 and is upregulated on CD3^+^ T cells in the peripheral blood, on CD8^+^ T cells in the aqueous humor, in oral ulcers on mononuclear cells in the skin and in intestinal lesions of patients with BD (12, 33, 38, 44). The highest CXCR3 expression on CD3^+^ T cells, which was significantly correlated with drastic IFN-γ production, was detected in patients with BD with concomitant pulmonary and central nervous system involvement, indicating the important roles of CXCR3 in the development of BD during the pulmonary and central nervous system symptoms (12). An inverse correlation between the percentage of CXCR3 expression in CD3^+^ T cells in peripheral blood and serum level of CXCL10 in patients with BD has been observed (38). CCR5 expression has been shown to be elevated in the tissue samples and peripheral blood lymphocytes of patients with BD irrespective of symptoms (12, 44). With potential as BD activity indicator, CXCR2 expressions on both total leukocytes and neutrophils in the relapsing phase has been shown to be significantly elevated in patients with ocular BD; low-dose prednisolone therapy reduces this expression on neutrophils (45). Nevertheless, more research is needed to assess the stability, reproducibility, sensitivity and specificity of these biomarker candidates, considering individual differences among patients with BD, to identify appropriate laboratory biomarkers.

## 4 Chemokines and chemokine receptors as therapeutic targets

Significant research has focused on strategies that target chemokines and chemokine receptors for various disease treatments. Till date, three chemokine/chemokine receptor-targeting drugs have been approved for clinical application. These include plerixafor (a CXCR4 antagonist), maraviroc (a CCR5 antagonist) and mogamulizumab (a CCR4 antagonist), respectively, for the mobilization of haematopoietic stem cells, inhibition of the human immunodeficiency virus and treatment of patients with relapsed or refractory adult T-cell leukaemia–lymphoma, mycosis fungoides and Sezary syndrome (46). As chemokines and chemokine receptors play a central role in the pathogenesis and development of BD, strategies that target chemokines and chemokine receptors potentially become a novel option for managing BD.

Poor understanding of chemokines and chemokine receptors in the pathogenesis of BD may have led to their neglect in therapeutic strategies. Thus, the progression of new treatment strategies in BD has been slow. Th1 interstitial migration is highly dependent on chemokine–chemokine receptor interaction and sensitive to the inhibition of G protein -coupled receptor signalling (47); these processes had shed light on the possible treatment strategies for Th1-mediated diseases. In addition, many strategies that target the chemokine–chemokine receptor system, including some small molecule blockers and antibodies [the CXCL10 antibody (MDX-1100), CCL2 antibody (Genzyme Techne) and CXCR4 inhibitor (AMD3100)] for common autoimmune diseases (such as rheumatoid arthritis, systemic sclerosis and systemic lupus erythematosus), have been developed and studied in preclinical and clinical trials with effective outcomes (9). The expression of CCR1 was found to decrease in a herpes simplex virus-induced mouse model of BD and upregulation of CCR1^+^ cells after colchicine and pentoxifylline treatment improved the symptoms of BD mice (48). Surprisingly, a preclinical study showed that the administration of anti-CCL3 antibody has therapeutic effects on BD and alleviates clinical manifestations in a mouse model by increasing the expression of CCR1, which is the receptor for CCL3 (48). Subsequent research should be focused on target selection, drug dosages, intervention time and adverse effects on patients with BD. In light of the above, treatment strategies that target chemokines and their receptors for BD treatment should be investigated extensively.

## 5 Conclusions and future prospects

Chemokines and chemokine receptors are important mediators in the development of BD as they regulate gene susceptibility and mediate cellular infiltration, activation and cascade amplification of inflammatory responses, suggesting their significant potential as laboratory biomarkers and therapeutic targets in the management of BD. However, a poor understanding of their roles in the pathogenesis of BD led to a lack of appreciation for their possible role in laboratory biomarkers and therapeutic strategies. Therapeutic strategies based on certain chemokines and their receptors, including anti-CXCL8, anti-CCL3, anti-CCR5 and anti-CXCR3, could become novel alternatives in BD treatment. Further investigations regarding the roles of chemokines and their receptors in BD and strategies that target the chemokine–chemokine receptor system are necessary.

## Author contributions

ZL and LC wrote the manuscript based on discussions with HZ and prepared the Table and Figure. YL revised and examined the manuscript. All authors contributed to the article and approved the submitted version.
